# A German perspective on the impact of socioeconomic status in diffuse large B-cell lymphoma

**DOI:** 10.1038/s41408-024-01158-9

**Published:** 2024-10-11

**Authors:** Susanne Ghandili, Judith Dierlamm, Carsten Bokemeyer, Henrik Kusche, Frederik Peters

**Affiliations:** 1 Department of Oncology, Hematology and Bone Marrow Transplantation with Section Pneumology, University Cancer Center Hamburg, University Medical Center Hamburg-Eppendorf, Martinistraße 52, 20246 Hamburg, Germany; 2 Hamburg Cancer Registry, Ministry of Science, Research, Equality and Districts, Free and Hanseatic City of Hamburg, Süderstraße 30, 20097 Hamburg, Germany

**Keywords:** Cancer epidemiology, Disease-free survival

## Abstract

The prognostic influence of socioeconomic status (SES) on the survival of diffuse large B-cell lymphoma (DLBCL) patients remains controversial. This observational study examines the potential impact of regional SES inequalities on overall survival (OS) among DLBCL patients in Germany. We analyzed data from the German nationwide population-based dataset spanning 2004-2019 sourced from the German Center for Cancer Registry Data (*n* = 49,465). The primary objective was to assess the 5-year OS among patients with low SES compared to those living in middle and high SES areas. SES was grouped according to quintiles of the German Index of Socioeconomic Deprivation, which summarized nine indicators covering aspects of regional education, employment, and income. DLBCL patients in low SES areas had significantly impaired 5-year OS compared to those in middle and high SES regions (59.2% vs. 61.8% vs. 64.1%, *p* < 0.0001). Yet, additionally accounting for regional premature mortality removed the impact of SES on survival (Hazard Ratio 0.94, 95% CI 0.87–1.01). Our findings indicate that the prognostic impact of socioeconomic deprivation on long-term survival is not due to variations in diagnosis and treatment of DLBCL itself but rather a higher comorbidity burden.

## Introduction

Currently, risk stratification and treatment decisions in diffuse large B-cell lymphoma (DLBCL) rely on the International Prognostic Index (IPI) [1–3]. In detail, the IPI compromises age, Eastern Cooperative Oncology Group (ECOG) performance status, lactate dehydrogenase level before treatment, disease stage defined as Ann Arbor, and the number of extranodal sides [1]. Depending on an IPI-based risk stratification, distinct first-line therapies, mainly combining a CD20-directed monoclonal antibody and, if suitable, a CD79b-directed antibody-drug conjugate with conventional anthracycline-based chemotherapy are discussed for the treatment of newly diagnosed DLBCL [3]. Since the IPI is based solely on biological factors, nonbiological factors like the patient’s socioeconomic status (SES) do not currently influence recommendations for treating DLBCL.

So far, the impact of SES on DLBCL patients’ survival has been controversially discussed [4–14]. Several population-based cohort studies from Denmark, the Netherlands, the United Kingdom, the United States, and Hong Kong have indicated that low SES decreases survival among patients with DLBCL [4–6, 8–11, 14]. Possible explanations focused on delayed or inadequate care because of inadequate health insurance [4, 6, 9, 15]. Individual factors that might be associated with reduced mortality have been discussed controversially, including employment status, income level, marital status, treatment in specialized hematological/oncological departments, type of insurance, and comorbidity burden [5, 6, 8, 12].

Previously, we observed in a case-control study examining the potential impact of neighbourhood SES inequalities on long-term survival among 2’134 DLBCL patients in the German metropole Hamburg, a vanishing impact of SES with an initial inferior OS for patients living in low SES neighbourhoods compared to the high SES group in the period from 1990-2003. This effect was observed solely in DLBCL patients within the initial period (1990-2003), with no consistent association between SES and mortality observed in the period 2004-2022, most likely explained by the implementation of rituximab-based immunochemotherapy, suggesting that adequate healthcare in a comprehensive health insurance setting may compensate for social disadvantage (yet unpublished results).

In this study, we now aim to investigate the potential influence of regional SES inequalities on long-term outcomes among DLBCL patients by conducting a large registry-based, nationwide German case-control study. To our knowledge, we here present the first study examining the impact of SES inequalities on a German nationwide basis.

## Materials/Subjects and Methods

### Data sources

This longitudinal study is based on an evaluated and harmonized pooled cancer registry dataset provided by the Centre for Cancer Registry Data (ZfKD) at the Robert Koch Institute for the years 2004-2019, covering the whole population of Germany with 84 million inhabitants. Cancer registration in Germany is implemented at the federal-state level. As a consequence, not all federal states collected complete data over the whole time period (North Rhine-Westphalia was fully covered since 2005, Hesse since 2007, Baden-Wuerttemberg since 2009 and the states located in East Germany, namely Berlin, Brandenburg, Mecklenburg-Western Pomerania, Saxony, Thuringia, Saxony-Anhalt up until 2013) [16]. To account for the differing levels of coverage, we carried out sensitivity analyses and included fixed effects for time and place in our models. All included cancer diagnoses were annotated with regional codes representing 16 federal states at the upper level, corresponding to the Nomenclature des Unités territoriales statistiques (NUTS) hierarchical level 1, and 401 regional districts, corresponding to the NUTS-2 level. Additionally, we extracted aggregated data on premature mortality for women and men at the regional level (NUTS-2) from the ongoing spatial observation of the Federal Institute for Research on Building, Urban Affairs, and Spatial Development. Data on regional deprivation at the NUTS-2 level per calendar year from 2004 until 2019 are based on the German Index of Socioeconomic Deprivation (GISD) of the Robert Koch Institute [17]. Finally, data on age- and sex-specific one-year death probabilities for the general population, denoted as background mortality, at the level of Germany were extracted from the Human Mortality Database of the Max Planck Institute for Demographic Research, University of California, Berkeley, and French Institute for Demographic Studies [18].

### Patients

We included all patients with a clinically and/or pathologically verified DLBCL diagnosis coded as “C83.3” according to the International Statistical Classification of Diseases, German Modification (ICD-10-GM) in combination with histology code “9680” of the International Classification of Diseases for Oncology, 3^rd^ Edition (ICD-O-3) between January 1st 2004 and December 31^st^, 2019 with follow-up until December 31^st^, 2019. Exclusion criteria were age at diagnosis below 18 or above 99 years, DLBCL located at the central nervous system (ICD-O-3 “C70”, “C71” or “C72”) and invalid district ID. Patients with a follow-up duration of less than three months were excluded to remove cases that entered the registry data only from information on death certificates, to avoid immortal time bias as reports up to three months of the initial report to the cancer registry were employed to specify the diagnosis and to focus on the impact of SES on long-term survival rather than on death due to acute conditions. We chose not to include patients diagnosed prior to 2004 to reduce the impact of heterogeneous treatment regimens (chemotherapy only vs. current rituximab-containing immunochemotherapy).

### Ethics approval and consent to participate

An assessment of the ethics committee and informed consent are not required based on federal law. As regulated in Section 6 Paragraph 2 of the HmbKrebsRG, the Hamburg Cancer Registry may participate in scientific studies on cancer, as long as no person is identifiable when the results are published and also uses the data from other state cancer registries for this purpose.

### Procedures

#### Study variables

At the patient level, study variables were place of residence (numbered from 1 to 16 according to federal state of the district), calendar year of diagnosis in years, age at diagnosis in years and in age groups (below 60 years, 60 years and above), sex (female, male) and a history of other primary tumors (none, one, two or more) and for each combination of calendar year, age and sex the corresponding level of background mortality, obtained from the general population in Germany.

At the district level, study variables were the presence of at least one certified hospital center in the district, the level of sex-specific premature mortality (deaths per 1’000 persons until age 70) as proxy for the comorbidity burden at the regional level and regional socioeconomic deprivation as proxy for socioeconomic status (SES). SES was measured using the GISD, which is an area-based deprivation index summarizing aggregated information on employees with a university degree, employees without education, school leavers without qualification, employment rate, unemployment rate, gross earnings, income tax, household income and debtor quota [17]. The annual GISD score was linked to each case in the cancer registry dataset by district id and year of diagnosis. Based on the quintiles of the distribution of the GISD, SES was grouped into low (5th quintile), middle (2nd to 4th quintile) and high (1st quintile). Earlier work confirmed the suitability of the index to study socioeconomic variations in cancer mortality in Germany [19].

### Outcomes

Primary outcome of the study was death within five years after date of diagnosis. Patients were followed until date of death, end of study period or 1’827 days, whichever occurred first.

### Statistical analysis

All analyses were conducted in R, Version 4.3.1. Categorial variables were summarized by counts and proportions and continuous variables by the median and the first and third quartile. Unadjusted overall survival by SES group was computed by Kaplan-Meier functions with logrank test. Cox proportional hazard models were used for assessing SES group differentials in 5-year survival adjusted for all study variables described above, except for premature mortality, which was added in a separate model to explore the potentially mediating impact of regional comorbidity burden. The proportional hazard assumption was assessed visually based on scaled Schoenfeld residuals. To account for unobserved variation over time and place of residence fixed effects were included based on dummy variables for each calendar year (2004-2019) and federal state [1–16]. Cluster robust standard errors were employed to take the correlation of observations within districts into account. As sensitivity analyses, the model was fitted separately for the calendar years 2004-2012 and 2013–2019, for all federal states other than the city registries in Hamburg, Bremen and Berlin and for all federal states excluding East Germany. The maps were generated using the R package *sf* for spatial data manipulation and the *tmap* package for thematic mapping and visualization. The spatial data, sourced from the VG2500 dataset of the Federal Agency for Cartography and Geodesy (BKG), was transformed to align with the EPSG:4314 coordinate reference system, ensuring consistency with the BKG’s reference system. Additionally, the data used adheres to the “Data licence Germany – attribution – Version 2.0”.

For reporting our results, we adhered to the Strengthening the Reporting of Observational Studies in Epidemiology (STROBE) statement [20].

## Results

**Fig. 1:**
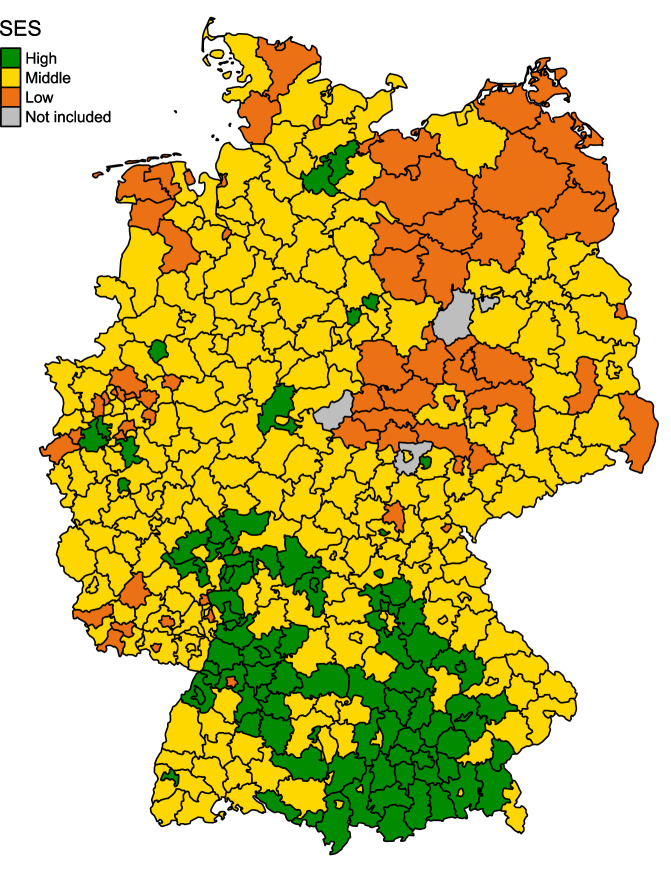
Geographical distribution of socioeconomic groups over 397 districts in Germany in 2019. SES Socioeconomic status.

**Fig. 2:**
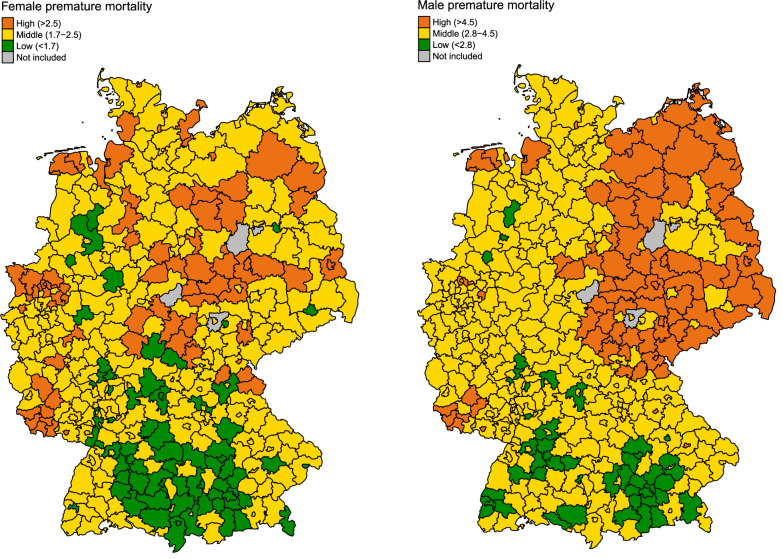
Geographical distribution of female and male premature mortality, defined as deaths before age 70 per 1000 inhabitants, over 397 districts in Germany in 2019.

Regions with low SES and a high level of premature female and male mortality were concentrated in the eastern districts of Germany, while regions with high SES and a low level of premature female and male mortality were concentrated in the southern districts of Germany (Figs. 1 and 2). Thus, for persons in East Germany, particularly for males, contextual conditions were inferior both for comorbidity/lifestyle and socioeconomic deprivation. Yet, the same was true for some regions of Western and Northern Germany.

**Fig. 3:**
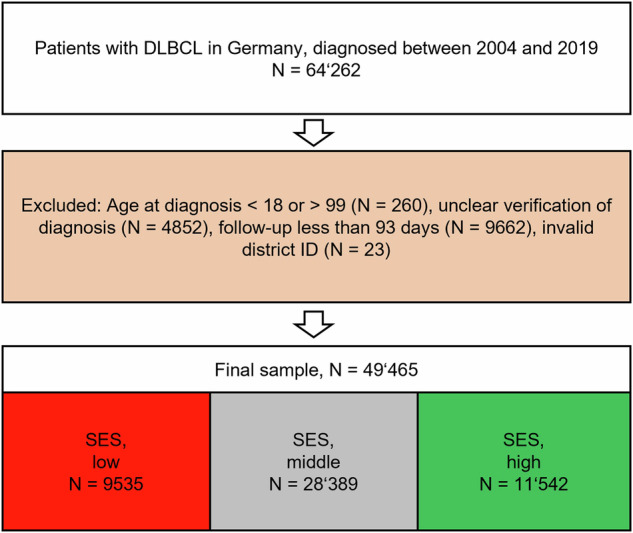
Flow chart. DLBCL diffuse large B-cell lymphoma, SES Socioeconomic status.

The final study cohort comprised 49’465 patients (low SES: 9’535, middle SES 28’389, high SES 11’154) with DLBCL diagnosis between 2004 and 2019 with a median age of 70 years, a median follow-up of 3.64 years and a proportion of 46.2% males for whom in total 16’680 deaths were observed in the study period (Fig. 3 and Table 1). The main reason for exclusion was a follow-up shorter than 93 days (9’662 cases of initially 64’262 representing 15% of the sample), indicating either cases based on a death certificate only, so-called DCO cases, terminally ill patients with a short lifespan or patients included at the end of 2019 (Fig. 3). About 34% of DLBCL patients in our sample died within five years, and the average background one-year death probability was 1.63%, while regional average premature mortality was 2.8 deaths per 1’000 persons, and about a third of patients lived in regions with at least one certified hospital center (Table 1).

**Table 1:** Baseline characteristics of patients with diffuse large B-cell lymphoma, stratified by regional socioeconomic status.

Germany, 2004-2019		Socioeconomic status		
	Total (*N* = 49‘465)	Low (*N* = 9535)	Middle (*N* = 28‘389)	High (*N* = 11‘541)
Age in years, median (Q1, Q3)	70 (59, 78)	70 (59, 78)	70 (59, 78)	70 (58, 78)
Age >=60 years, (%)	36,555 (73.9)	7079 (74.2)	21022 (74.0)	8454 (73.3)
Background one-year death probability, in percent, median (Q1, Q3)	1.63 (0.61, 3.54)	1.56 (0.62, 3.44)	1.64 (0.62, 3.57)	1.61 (0.58, 3.57)
Gender, male (%)	22,866 (46.2)	4554 (47.8)	12972 (45.7)	5340 (46.3)
Other prior primary tumor (%)				
None	42,039 (85.0)	7988 (83.8)	23925 (84.3)	10126 (87.7)
One	6308 (12.8)	1296 (13.6)	3769 (13.3)	1243 (10.8)
Two or more	1118 (2.3)	251 (2.6)	695 (2.4)	172 (1.5)
At least one certified hospital center in district (%)	15,853 (32.0)	3345 (35.1)	7904 (27.8)	4604 (39.9)
Premature mortality (deaths per 1000 persons until age 70 in district) (%)	2.80 (2.12, 3.53)	3.43 (2.48, 4.57)	2.93 (2.19, 3.64)	2.36 (1.73, 2.92)
Median follow-up in years (Q1, Q3)	3.46 (1.21, 5.00)	4.21 (1.25, 5.00)	3.38 (1.17, 5.00)	3.29 (1.21, 5.00)
Deaths within five years (%)	16,680 (33.7)	3623 (38.0)	9497 (33.5)	3560 (30.8)

*Q1*: First quartile, *Q3* Third quartile.

Compared to patients in regions with high SES, those in regions with low SES had a slightly higher share of males (47.8% versus 46.3%), more often reported earlier primary tumors (16.2% versus 12.3%), and lived less often in regions with a certified hospital center (35.1% versus 39.9%) while at the same time, their regional level of premature mortality was higher (3.43 deaths/1’000 persons versus 2.36 deaths /1’000 persons; Table 1).

**Fig. 4:**
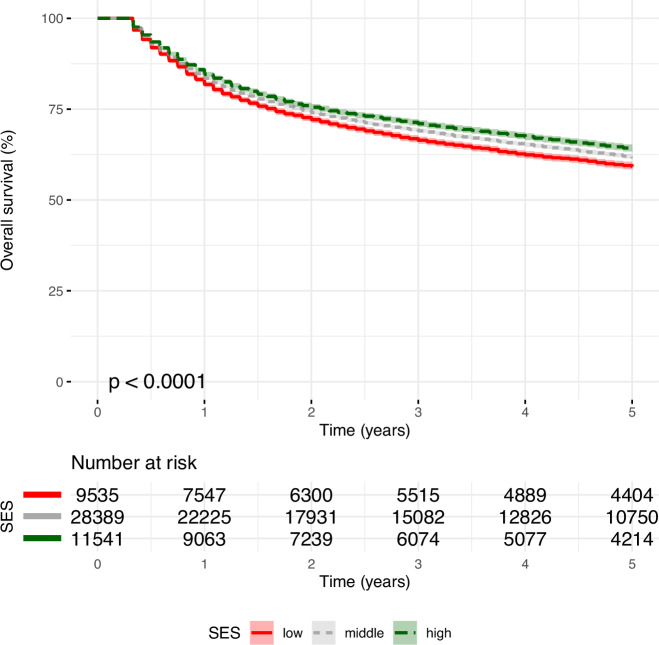
Five-year overall survival of diffuse large B-cell lymphoma patients, stratified by socioeconomic status. SES Socioeconomic status.

**Fig. 5:**
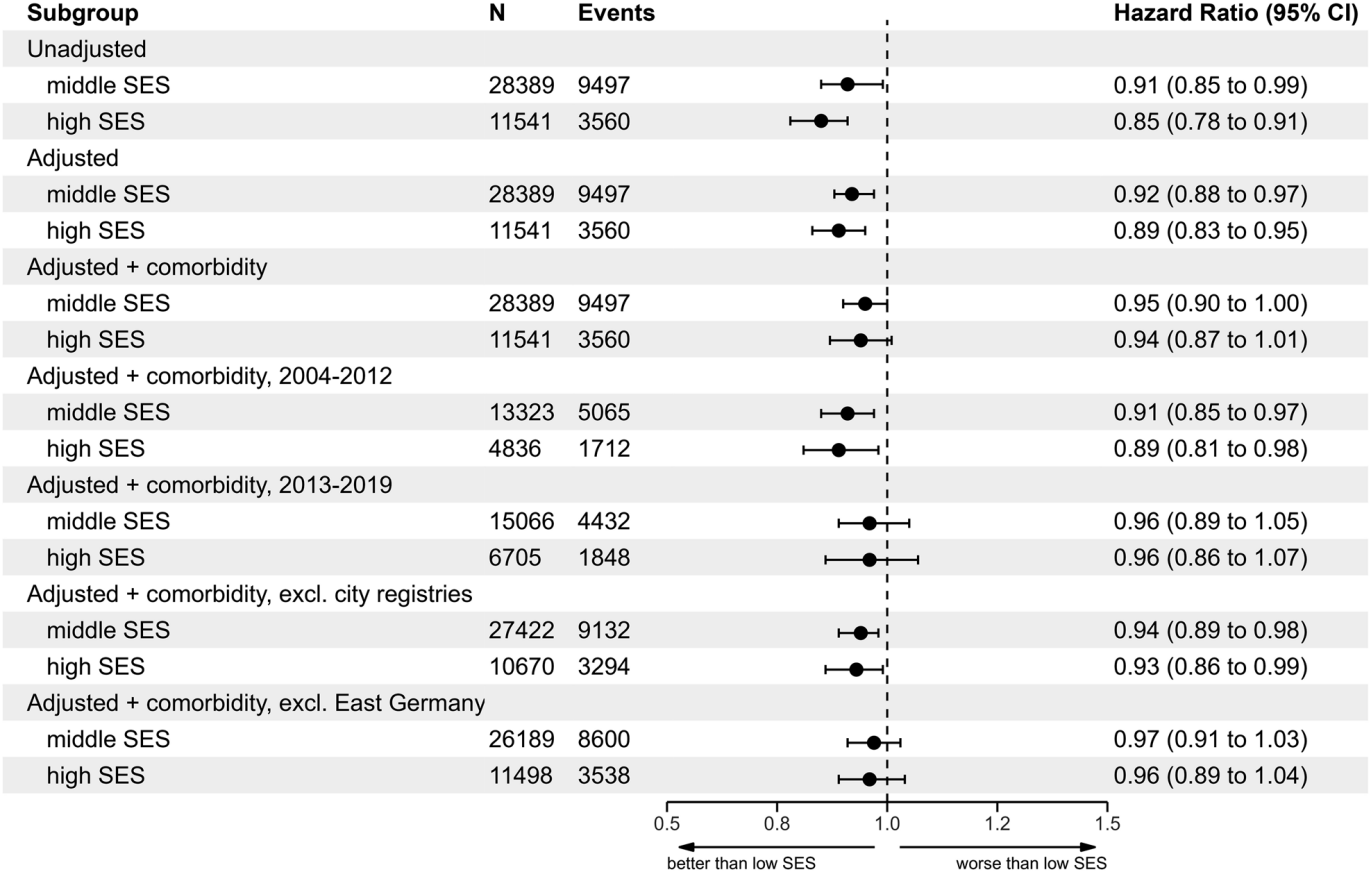
Estimated hazard ratios with 95% confidence intervals for the association of socioeconomic status (Reference group: low SES) and death within 5 years in patients with diffuse large B-cell lymphoma (*N* = 49‘465); SES Socioeconomic status.

Unadjusted 5-year survival differed slightly but significantly (*P* value < 0.001) and stable over follow-up time between SES groups ranging from about 59% in patients living in regions with low SES to about 62% in patients living in regions with high SES (Fig. 4). Compared to the group with low SES, the unadjusted hazard ratio (HR) in the middle SES group was 0.91 (95% confidence interval: 0.85-0.99) and 0.85 (95% confidence interval 0.78-0.91) in the high SES group (Fig. 5).

After adjusting for patient characteristics, these differentials remained largely similar at a hazard ratio of 0.92 (95% confidence interval: 0.88-0.97) and 0.89 (95% confidence interval 0.83-0.95). Yet, after additionally taking variations in regional premature mortality into account, differences between SES groups diminished (Adjusted HR for middle SES 0.96, 95% confidence interval 0.90-1.00, and adjusted HR for high SES 0.94, 95% confidence interval 0.87-1.01).

Sensitivity analyses revealed that even after adjusting for all confounders, in the subgroup of patients diagnosed in 2004-2012, significant differentials remained between SES groups, which diminished almost completely in the most recent period covering 2013-2019 (Fig. 5). Excluding city registries (Hamburg, Bremen, and Berlin) slightly increased SES group differentials, and excluding all districts in East Germany slightly decreased SES differentials. Yet, taken together, there were only small differences among the adjusted models, and all confidence intervals overlapped with each other. Based on the point estimates, the relative difference in mortality risk for patients in regions with high SES compared to low SES ranged from 11% to 4% and from 19% to -7% based on 95% confidence intervals.

## Discussion

By analyzing data from a German nationwide population-based dataset including 49’465 patients, derived from the German Center for Cancer Registry Data, and spanning the last 15 years, we observed that DLBCL patients living in low SES areas had significantly impaired 5-year OS compared to those living in middle and high SES regions. However, after additionally accounting for regional premature mortality, the impact of SES on survival was abrogated, leading to the conclusion that the prognostic impact of socioeconomic deprivation on long-term survival in patients with DLBCL is not a result of variations in diagnosis and treatment of DLBCL itself but rather a result of an inferior health-related lifestyle and higher comorbidity burden in patients living in low SES regions. To the best of our knowledge, we here presented not only the first German nationwide study analyzing the impact of regional SES on OS in DLBCL patients but also analyzed one of the largest study populations worldwide.

With a 9% and 15% reduced mortality risk in patients living in middle and high SES areas, respectively, compared to those living in low SES areas, our results basically align with those previously reported by Frederiksen et al., Tao and colleagues, Lee et al., and Smith et al. from Denmark, the United States, Hong Kong, and England observing excess mortality in DLBCL patients living in low SES areas [4, 6, 9, 10]. In fact, Tao et al. reported the results of a large observational study on 33’032 DLBCL patients living in the U.S. state of California, concluding that DLBCL patients living in the lowest SES neighborhoods had a 25% and 37% greater risk of death from DLBCL and all-cause, respectively, compared with those living in the highest SES neighborhoods. Possible explanations focused on inadequate insurance coverage in the United States [6]. Similar to our conclusion, an analysis of the Swedish lymphoma registry reported that comorbidities defined as a Charlson Comorbidity index of more than one comorbidity are associated with an inferior outcome in DLBCL patients, mainly explained by the authors by a lower likelihood of receiving curative intended treatments. However, the authors also reported that comorbid patients treated with curative intent had no impaired lymphoma-specific survival but rather an inferior overall survival [21]. In addition, Dhakal et al. analyzed data from the U.S. National Cancer Database, including 185’183 DLBCL patients, observing that a lower Charlson Comorbidity index was associated with an improved 1-month mortality and OS [8]. Moreover, analysis from Denmark, France, and the North-West of the Netherlands reported that mild and severe comorbidities were independently associated with unfavorable relative survival and an increased mortality risk in DLBCL patients even in the absence of directly SES-associated survival differences [7, 12, 14, 22].

This study uncovered that SES does not directly influence DLBCL-specific survival but rather indirectly by elevating risks for other diseases. This might be viewed as empirical evidence for the theory of “fundamental causes”, arguing that people with low SES generally lack resources, knowledge, and abilities to avoid general disease risks over the course of their lives [23]. Thus, we assume that the prevention of lifestyle-related risk factors like smoking, and obesity might be a strategy to reduce the comorbidity burden and to compensate for socioeconomic deprivation. However, the relevance of other lifestyle-related factors, such as alcohol consumption, use of health care, and working and housing conditions, remain yet unaddressed [24]. Since DLBCL typically affects individuals at a median age of 70 years, with a comorbidity burden ranging between 6 to 26%; thus we assume that standardized geriatric assessments might serve as a systematical evaluation tool for comorbidities, enabling the early development of interdisciplinary concepts for diagnosis and treatment of relevant comorbidities, e.g., heart failure or impaired kidney function to finally optimize cancer-directed treatment and reduce potential therapy toxicities in parallel with cancer-directed treatment [3, 21, 22, 25].

Furthermore, the fact that there were no measurable SES differentials in a country with universal health care coverage suggests that such unwarranted variations could be perhaps completely avoided if all patients receive access to optimal guideline-directed therapy. The example of huge and even widening SES differentials over time in the US impressively demonstrates that indeed not health care expenditures but perhaps rather healthcare coverage may play a central role for diseases with intensive, costly, and complex treatment schemes [6].

This epidemiological study is based on cancer registry data, containing general information on patient characteristics and tumor diagnosis but lacking more specific prognostic variables such as Ann-Arbour stage or IPI, hospital volume and certification, information on treatment as well as lifestyle and comorbidities, but also the economic situation and educational attainment at the patient level. Thus, it might be the case that SES indeed has a considerable prognostic impact on DLBCL-specific survival if all these aspects had been considered. However, this potential residual confounding is mitigated by our study design explicitly considering a range of contextual factors, namely premature mortality as a proxy for lifestyle and comorbidity and the proximity of a certified center as a proxy for quality of treatment. Moreover, by including fixed effects for calendar year and federate state into the models, we indirectly adjusted for unobserved patient characteristics that change over time or differ between places or are related to the fact that not all local registries provided full coverage of DLBCL patients over the whole study time or other aspects of cancer registration that changed over time. We are therefore confident that our results based on a large and country-wide cohort sample are robust, which is confirmed by a range of sensitivity analyses. Another limitation of the study is that the central conclusions are based on a single endpoint only, namely all-cause mortality since more specific data on cancer progression or cause of death were not available in the registry data. However, general age-, sex- and calendar year-specific mortality were added in all models to consider the background risk for death, which allows us to indirectly estimate disease-specific mortality and to take a higher comorbidity burden associated with higher age and male sex into account. In addition, it must be mentioned that 15% of our initial sample sizes had to be excluded due to insufficient follow-up duration, e.g. because they are based on death certificate only without more detailed information than age and sex. It would be a worthwhile question for further research to disentangle the influences of SES, acute conditions and frailty on short-term survival in DLBCL patients. However, since early mortality within the first three months after initial DLBCL diagnosis is relatively low, our main conclusions regarding long-term survival are likely less impacted.

Further studies based on more detailed data at the patient level should aim at verifying our central hypothesis that existing SES differentials among DLBCL patients are explained by factors not related to the diagnosis and treatment of the disease itself but rather by other coexisting risk factors and diseases. However, from a nationwide perspective, even though a comprehensive health insurance setting exists, socioeconomic deprivation is related to a higher comorbidity burden and is translating into significant survival differences. For this reason, we conclude that although SES is not a prognostic factor for survival in DLBCL patients, medical guidelines may emphasize the need for tailoring treatment to the specific vulnerability caused by comorbidities in patients with socioeconomic deprivation. Further research is needed to understand better the complex relationship between SES, comorbidity burden, and survival in DLBCL patients.

## Data Availability

The data included in the study were analyzed in the protected environment of the Hamburg Cancer Registry. Due to data protection and security regulations, the sensitive patient data cannot be made publicly available. In general, however, data for scientific projects can be requested from the Hamburg Cancer Registry. All analysis scripts can be made available on request from the corresponding author.
